# Tropical rivers face severe oxygen stress caused by heat and organic matter: new evidence by stable isotopes

**DOI:** 10.1038/s41598-026-65862-1

**Published:** 2026-08-10

**Authors:** Sachintha Senarathne, Robert van Geldern, Rohana Chandrajith, Johannes A. C. Barth

**Affiliations:** 1 Department Geographie und Geowissenschaften, GeoZentrum Nordbayern, Friedrich-Alexander-Universität Erlangen-Nürnberg (FAU), Schlossgarten 5, 91054 Erlangen, Germany; 2 Department of Geology, Faculty of Science, University of Peradeniya, Peradeniya, Sri Lanka

**Keywords:** Dissolved oxygen, Stable isotopes, Photosynthesis, Respiration, Organic matter, Biogeochemistry, Climate sciences, Ecology, Ecology, Environmental sciences

## Abstract

Many rivers worldwide exhibit declining dissolved oxygen (DO) concentrations due to changes in hydroclimatic conditions and land use. Our study shows a typical tropical river in Sri Lanka (the Deduru Oya) with DO concentrations between 1.5 and 9.7 mg L^−1^. In this dataset, almost 80% of all collected water samples were undersaturated. In addition, rice paddies and extensive surface runoff during the monsoon season added organic matter, enhancing respiration and DO consumption. DO concentrations and associated stable isotopes (expressed as δ^18^O_DO_ in ‰) provided new insights into how temperature, respiration, and photosynthesis interact and control the aqueous DO cycle. Values of δ^18^O_DO_ varied from + 13.2 to + 27.7 ‰. Most headwater streams were within the range for atmospheric equilibrium (+ 24.6 ‰ ± 0.4 ‰). Further, downstream isotope values and DO saturations confirmed that the river became dominated by respiration. Only connected reservoirs and their nearby downstream sections showed influences by photosynthesis with typical isotope values lower than + 24.6 ‰. The new dataset is representative for tropical rivers and outlines critical zones and time periods that offer improvement of water management strategies and related ecosystem functioning.

**Supplementary Information:**

The online version contains supplementary material available at 10.1038/s41598-026-65862-1.

## Introduction

DO is a key indicator of ecological quality and arguably remains the most sensitive tool to quantify and monitor ecosystem water quality^1–5^. Water temperature is an important parameter that impacts DO saturations via Henry’s law^6^. Further temperature-related influences span physical, chemical, and biological functions that drive metabolic rates, nutrient cycles, and primary productivity^7,8^.

Increases in water temperature due to climate change correlate with a decline in DO concentrations in many surface waters worldwide^9,10^. Analyses of Landsat observations and climate data over the past four decades indicate that global surface waters have experienced a strong decline in DO of 0.045 mg L^−1^ per decade^11^. This temperature-driven DO decline is, for instance, pronounced in the Parana Plateau, Southeast United States (US), in Brazilian highlands, Central Europe and India^9–12^. Approximately 8.6% of global rivers experience declines in DO concentrations in response to rising air temperatures, with half of these rivers found in tropical regions^11^.

Furthermore, DO dynamics in climate transition zones are more sensitive to temperature fluctuations^13^. For instance, Indian rivers experienced a more substantial oxygen depletion, exceeding 0.2 mg L^−1^ per decade, whereas rivers in the United States and Central Europe showed a decrease of only 0.038 mg L^−1^ per decade^11,12^. Despite sensitivity and complexity, tropical freshwater systems remain underrepresented in investigations of low-oxygen events when compared to temperate, lake or marine systems^14,15^.

Tropical rivers are important freshwater ecosystems in the world, as they encompass nearly 19% of the global land area and transport 66% of global freshwater and 59% of organic carbon into the oceans^16–19^. These tropical freshwater ecosystems are heavily affected by climate change scenarios. In particular, regions such as tropical South America and South Asia show increasing tendencies towards drying and droughts^16,20^.

In addition to influences by temperature, deterioration of surface water quality by organic matter input is another important factor^15,21,22^. A large proportion of the organic matter in natural waters is reported as dissolved organic carbon (DOC) and originates predominantly from terrestrial organic matter^23^. Particulate organic carbon (POC) is another important carbon phase in river organic matter^24^. Organic matter contents can be elevated in tropical rivers due to enhanced input by surface runoff and interflow during monsoon and additional storm events. For instance, tropical rivers such as the Mekong and Ganges exhibit four- to sixfold increases in POC concentrations during the high-water period when compared to the low-water periods^25^. In many tropical rivers, organic matter fluxes are further controlled by land-use. For instance, river-associated rice paddies are a common practice along many tropical river systems^26^. These agricultural practices further enhance organic matter, which is considered a stressor of low DO concentrations in aquatic environments^15,27^. In addition, many tropical rivers lack efficient sewage treatment, a factor that may increase organic matter input and accelerate DO consumption^28,29^. Furthermore, the damming of rivers for drinking water, irrigation, and power generation impacts rivers worldwide^30–33^. Such constructions usually enhance photosynthesis by slower flow velocities, clearer waters and nutrient availability^34–36^. Such geo-climatic and anthropogenic alterations underscore the challenge of identifying the key drivers of DO sources and sinks in aquatic systems. In addition to concentration measurements, an additional tool to map DO dynamics is measurements of its stable isotope ratios (^18^O/^16^O, expressed as δ^18^O_DO_ in permille [‰])^4,37–40^.

DO concentrations have been studied in several tropical rivers^3,15,41–43^. Generally, most tropical rivers show spatial and temporal variations in DO, often with critically low concentrations of less than 3.0 mg L^−1^ that mark hypoxia^44,45^. However, most of these studies primarily focus on the spatial variability of DO and its relationships with and sensitivities to socio-environmental factors, such as urbanisation, agricultural land-use changes and associated water management^3,15,41^. These studies identified a lack of spatial and temporal data coverage as a particularly common issue in small river studies, representing major research limitations that could be improved in future studies^41,43^. Another shortcoming of considering only concentration is that they are unable to outline sources or sinks of DO.

Here we present a new study of DO concentrations and their δ^18^O_DO_, in a tropical river, the Deduru Oya in Sri Lanka. This study adds new insights by integrating river water and DO isotope data and outlines atmospheric exchange (A), photosynthesis (P) and respiration (R) as key controls on DO concentrations. Together with determined relative influences by R and P, our findings provide a deeper understanding of DO and the mechanisms that control its input and loss for improved ecosystem management.

### Study area

**Fig. 1:**
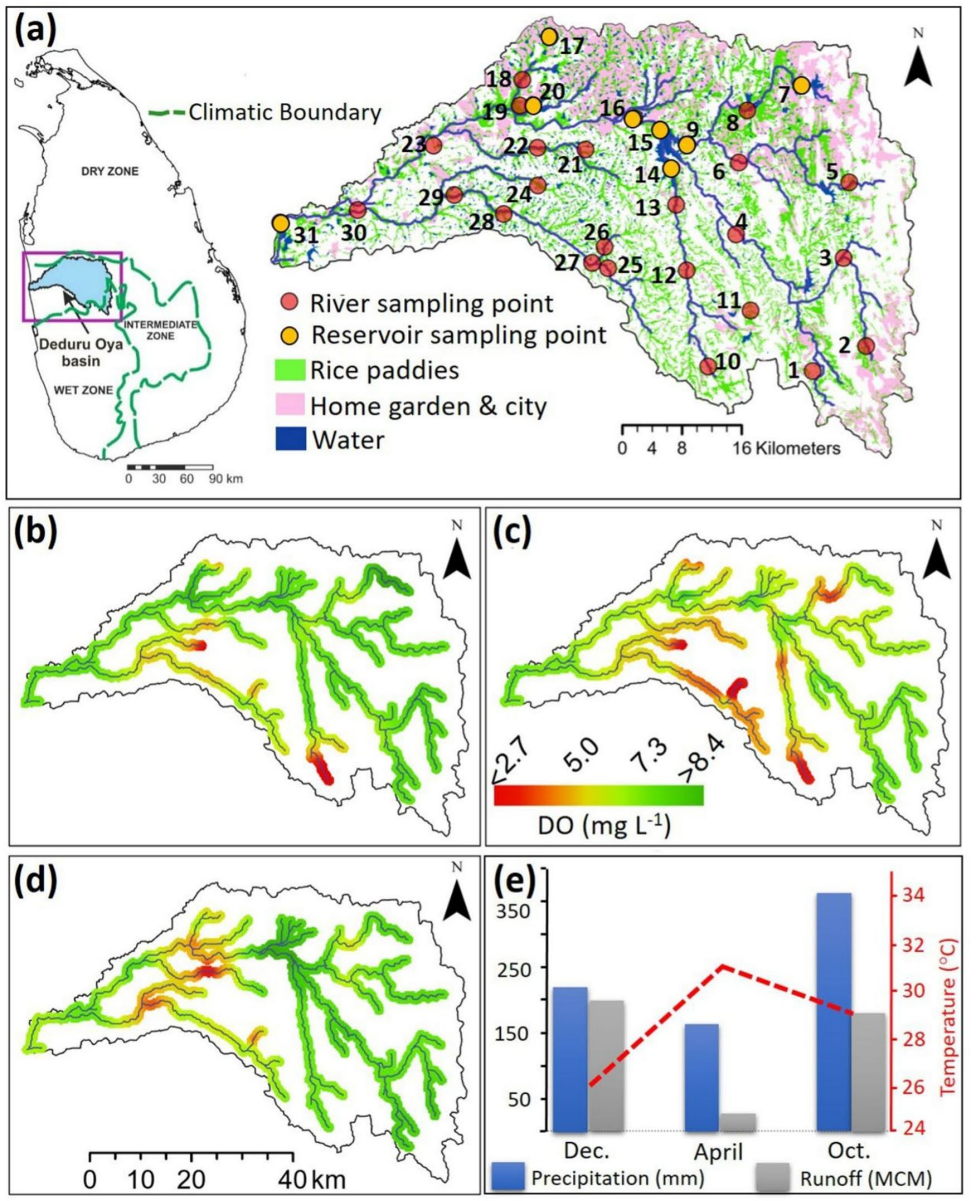
(**a**) Overview of Sri Lanka (left) and land use in the Deduru Oya Basin with sampling locations. Subfigures (**b**–**d**) show spatial variations of DO concentration (mg L^−1^) in December (**b**), April (**c**), and October (**d**). Subfigure (**e**) shows cumulative monthly precipitation and runoff, along with the average water temperature for each sampling campaign. Precipitation is in mm and runoff in million cubic meters MCM. The overview map was created via ArcGIS pro version 3.1 (https://www.esri.com) with raster data from United States Geological Survey (USGS) Earth Explorer (United States Geological Survey, 2024) via https://earthexplorer.usgs.gov. Land use data from the Survey Department of Sri Lanka, (2022) via https://survey.gov.lk.

Sri Lanka lies 800 km north of the equator. Its climate is mainly controlled by the movement of the Intertropical Convergence Zone. The mean ambient air temperature varies from 9 to 34 °C, depending on elevation^46^. The island has two major monsoon events, namely the south-west monsoon (SWM) from May to September and the north-east monsoon (NEM) from December to February. Average annual rainfall in Sri Lanka ranges from 90 cm to over 550 cm, and the country is divided into three major climatic zones (wet, intermediate, and dry)^47^ (Fig. 1a). Extreme climatic events can impose severe pressures on water resources in Sri Lanka. For instance, between 1980 and 2015, the island experienced 20 extreme droughts and precipitation events^48^, and the most recent tropical cyclone (Ditwa) in November 2025 accounted for almost 15.8% of the island’s average annual rainfall in only 3 days^49^. Such events, together with regular monsoons, mobilise large amounts of organic matter into rivers via surface runoff and interflow.

The total land area of Sri Lanka is 65,610 km^2^; of this, 53.88% consists of agricultural land, 30.9% of forest areas, and 0.92% of built-up areas, while the remaining 14.3% is occupied by water bodies and other forms of vegetation^46^. Further, the island has over 18,000 minor reservoirs, including 309 major irrigation ponds, across its 103 river basins that are mainly used for supply to rice paddies^50^. These reservoirs cover 2.45% of the total land area of the country. Of these reservoirs, 54.9% (882 km^2^) are permanent, and 45.1% (726 km^2^) are present seasonally^50^. So far, it remains unclear if and how hydrological, ecological, and geomorphological connectivities of these reservoirs affect rivers in Sri Lanka.

The Deduru Oya River Basin is the fourth largest catchment in the country, with an area of 2616 km^2^^51^. The headwaters lie at an altitude of 850 m above sea level, and the river flows for 115 km through all three climatic zones. Nearly 94% of the catchment area lies in the intermediate zone with moderate rainfall (170 cm yr^−1^)^52^ (Fig. 1a). The long-term average annual precipitation in the Deduru Oya Basin is 160 cm, with strong spatio-temporal variations. For instance, in the upstream wet zone, annual rainfall reaches up to 260 cm, while it ranges around 110 cm in the downstream intermediate zone. Moreover, the Deduru Oya Basin often experiences flash floods during the monsoon season. On the other hand, the catchment also experiences long, dry, and hot spells (> 28 °C) in between monsoons^53^. The runoff of the basin ranged from 39.8 to 63.7% of the incoming precipitation during the dry season and from 50.7 to 80.5% of the incoming precipitation during the rainy season^54^. The Deduru Oya Basin hosts seven major water reservoirs that connect to the river and numerous small water retention systems with coconut and irrigated rice paddies as the major associated vegetation types^53^. Rice cultivation is carried out in two growing seasons per year with field preparation in April and October^55^.

## Materials and methods

### Field methods

A total of 31 sites along the Deduru Oya River, its major tributaries and reservoirs were sampled in December 2022 and in April and October of 2023 (Fig. 1a). These sampling campaigns cover monsoon and inter-monsoon events as well as cropping seasons.

River samples were collected using a depth water sampler about 50 cm below the water surface to minimize the effects of rainfall and evaporation. In-situ physicochemical parameters (temperature, DO, and saturation (DO %)) were measured with a multi-parameter kit (HACH HQ40d and Sension-5). The 1σ standard deviation of the HACH™ instrument was ± 0.07 °C for temperature, ± 0.1 mg L^−1^ for DO, and ± 0.42% for DO %.

We also collected samples for analyses of oxygen stable isotopes of DO (δ^18^O_DO_) and water isotopes (δ^18^O_H2O_) as necessary inputs for the calculation of R/P ratios. All samples were filtered through 0.45-µm pore size disposable polyethersulfone syringe filters. For δ^18^O_DO_, pre-filtered samples were filled into 12-mL vials that were pre-poisoned with 20 μL of a supersaturated mercuric chloride (HgCl_2_) solution to avoid secondary microbial processing. Samples for δ^18^O_H20_ were filtered into separate 12-mL vials. All samples were preserved in the dark at 4 °C until analyses.

Water samples for DOC concentrations were filtered through 0.45 μm pore size disposable polyethersulfone (PES) syringe filters into 40 mL amber glass vials (so-called EPA vials) with a butyl rubber/PTFE septum with the butyl rubber side towards the sample. These samples were poisoned with 20 µL of a saturated HgCl_2_ to avoid secondary biological activity. Water samples for POC were collected in 500-mL Nalgene bottles.

### Laboratory methods

Values of δ^18^O_DO_ were determined with a modified automated equilibration system (Gasbench II, Thermo Fisher Scientific™) coupled in a continuous-flow mode to a DELTA V Advantage isotope ratio mass spectrometer by Thermo Fisher Scientific™. Details of the analytical procedure are available in Barth et al.^37^ and Wassenaar and Koehler^56^. In brief, a 3-mL helium headspace was created in all sample vials with a double-hole needle. Vials were then shaken for 30 min on an orbital shaker at 250 rotations per minute to extract all dissolved gases into the headspace. The extracted O_2(g)_ in the headspace was then separated from nitrogen (N_2_) and argon (Ar) by a gas chromatographic column (CPMolsieve 5 Å, 25 m capillary length, 0.53 mm outer diameter, 0.05 mm inner diameter; Agilent™, Santa Clara, CA, USA) before introduction into the isotope ratio mass spectrometer for analysis.

Water isotopes (δ^18^O_H2O_) were determined by wavelength-scanned cavity ring-down spectroscopy (Picarro L1102-i). Quality controls were carried out according to van Geldern and Barth^57^.

All stable isotope values were expressed in the standard δ per-mille (‰) notation with respect to the Vienna Standard Mean Ocean Water (V-SMOW) according to:

1 $$\updelta \left( \permil \right) = \left( {\frac{{{\mathrm{R}}_{{{\mathrm{sample}}}} }}{{{\mathrm{R}}_{{\mathrm{V-SMOW}}} }} - 1} \right)*1000$$

where R is the molar ratio of heavy to light isotopes (i.e., ^18^O/^16^O) in both samples and V‐SMOW. The ^18^O/^16^O ratio of this standard equals 2005.20 ± 0.43 ppm^58^.

Results are reported as averages of duplicate analyses, and external reproducibility (1-σ) was better than ± 0.2 ‰ for δ^18^O_DO_ and 0.1 ‰ for δ^18^O_H2O_.

### Isotope calculations

Ratios of respiration to photosynthesis (R/P) were calculated according to the following equation by Quay et al.^59^:

2 $$\frac{{\mathrm{R}}}{{\mathrm{P}}} = \frac{{\left( {{ }^{18/16} {\mathrm{O}}_{{\mathrm{w}}} *\upalpha _{{\mathrm{p}}} { } - { }^{18/16} {\mathrm{O}}_{{\mathrm{g}}} } \right)}}{{\left( {{ }^{18/16} {\mathrm{O}}*\upalpha _{{\mathrm{r}}} { } - { }^{18/16} {\mathrm{O}}_{{\mathrm{g}}} } \right)}}$$

where ^18/16^O_w_ and ^18/16^O are the isotope ratios of oxygen in water and in DO. Fractionation factors for photosynthesis (α_p_) and respiration (α_r_) were assumed to be 1.000 and 0.982^59–61^.

The term ^18/16^O_g_ in Eq. (2) represents the isotope ratio of net air–water fluxes and is calculated as follows:

3 $${ }^{18/16} {\mathrm{O}}_{{\mathrm{g}}} = { }\frac{{\upalpha _{{\mathrm{g}}} *{ }\left( {{ }^{18/16} {\mathrm{O}}_{{\mathrm{a}}} *\upalpha _{{\mathrm{s}}} - \frac{{{\mathrm{O}}_{2} }}{{{\mathrm{O}}_{{2{\mathrm{s}}}} }}*\;^{18/16} {\mathrm{O}}} \right)}}{{\left( {1 - \frac{{{\mathrm{O}}_{2} }}{{{\mathrm{O}}_{{2{\mathrm{s}}}} }}} \right)}}$$

where ^18/16^O_α_ is the atmospheric oxygen isotope ratio of + 23.9 ‰^38^, and α_g_ is the fractionation factor of gas transfer velocities with 0.9972^62^. The fractionation factor for oxygen dissolution in water (α_s_) was assumed to be 1.0007, as calculated by Benson and Krause^63^. Further, O_2_ represents the DO concentration in the sample, and O_2S_ represents the maximum temperature-dependent equilibrium DO concentration, as calculated by Benson and Krause^63^:

4 $$\begin{aligned} {\mathrm{O}}_{{2{\mathrm{s}}}} { }\left( {{\text{mg L}}^{ - 1} } \right) & = {\mathrm{exp}}\left[ { - 139.34411 + { }\frac{{1.575701*10^{5} }}{{\mathrm{T}}} - \frac{{6.642308*{ }10^{7} }}{{{\mathrm{T}}^{2} }}} \right. \\ & \quad \left. { + \frac{{1.243800*{ }10^{10} }}{{{\mathrm{T}}^{3} }} - \frac{{8.621919*{ }10^{11} }}{{{\mathrm{T}}^{4} }}} \right] \\ \end{aligned}$$

where T is the measured water temperature in kelvin. Multiplication of $\frac{{O_{2} }}{{O_{2s} }}$ by 100 yields DO saturation (DO %) as measured in the field with a good match to calculated values.

DOC concentrations were measured using a two-step phosphoric acid and persulfate wet oxidation procedure on a TIC-TOC analyser (Aurora 1030W, OI Analytical, College Station, Texas, USA). This is operated in continuous-flow mode and coupled to an isotope-ratio mass spectrometer (IRMS, Delta V plus, Thermo Scientific, Bremen, Germany). Further details of the analytical procedure are described in St-Jean^64^ and van Geldern et al.^65^.

POC samples were filtered through pre-heated (400 °C for 4 h) and pre-weighed glass fibre filter papers with a pore size of 0.4 μm. After filtration, the filters were freeze-dried and reweighed with the retained material. POC content was determined by calculating the difference between the weight of the loaded and dried filters and their measured carbon content. The loaded filters were pulverised in a carbon-free mortar using a ball mill (Retsch CryoMill). To remove carbonate particles, pulverised samples were dried at 60 °C and fumigated with concentrated HCl in a desiccator for 24 h. The prepared material was weighed into tin capsules and analysed using a Costech Elemental Analyser (ECS 4010, Costech International, Pioltello, Italy) in continuous-flow mode coupled to a Thermo Scientific Delta V Plus IRMS. DOC and POC concentration measurements had relative standard deviations (RSD) better than ± 5%.

The data set was analysed using the nonparametric Kruskal–Wallis test to determine whether significant differences exist between sampling campaigns. All statistical analyses were conducted in RStudio 2025 (version 4.5.0). Topographical features and interpolation maps were graphically illustrated in ArcGIS Pro (version 3.1). For interpolation, the inverse distance weighting (IDW) method was applied. This method reduces computational and modelling efforts and assumes that the influence of the mapped variables decreases with increasing distance from their sampling points^66,67^.

## Results

Surface water temperature in the Deduru Oya River ranged from 25 to 34 °C with pronounced seasonal fluctuations (*p* < 0.05). Highest values occurred in April and ranged from 27 to 34 °C. In October, they ranged from 26 to 31 °C and in December from 25 to 31 °C (Table S1, supplementary material).

Measured DO concentrations ranged from 1.5 to 9.7 mg L^−1^ in the entire dataset and showed clear seasonal variations between April (inter-monsoon) and December (monsoon) (*p* < 0.05). In April, DO concentrations were lowest, ranging from 1.5 to 8.4 mg L^−1^. In October, they ranged from 3.5 to 9.6 mg L^−1^ and in December from 2.0 to 9.7 mg L^−1^ (Table S1). These trends negatively correlated with temperature and were less clearly related to precipitation and runoff (Fig. 1e). DO concentrations also showed clear spatial variations along the river and its tributaries. These spatial trends were consistently present in December (Fig. 1b) and October (Fig. 1d), but variations were more pronounced in April (Fig. 1c). Two tributaries in the southern and central parts of the catchment often showed critically low DO (< 5 mg L^−1^).

Unlike DO concentrations, DO saturation levels (DO %) in surface water showed less seasonal variation (*p* > 0.05). Nonetheless, we found large ranges of DO % levels from 20 to 120% in April, from 46 to 124% in October, and from 26 to 128% in December (Table S1). Most river samples showed DO undersaturation (median = 88%), whereas most reservoir samples were oversaturated (median = 103%).

**Fig. 2:**
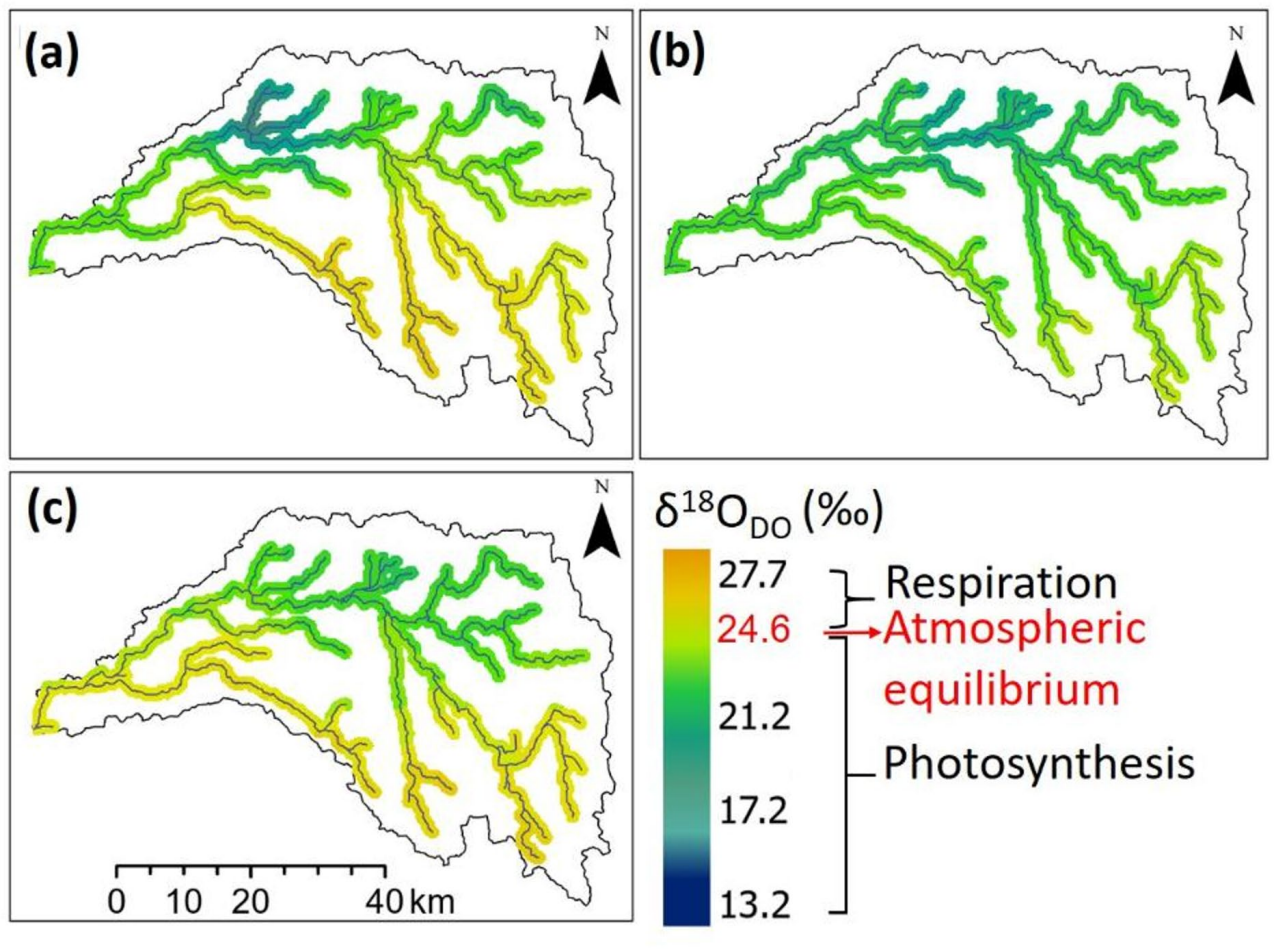
Spatial variations of δ^18^O_DO_ in December (**a**), April (**b**), and October (**c**).

DO stable isotope (δ^18^O_DO_) values ranged from + 13.2 ‰ to + 27.7 ‰ (Table S1). Reservoir water samples showed lower δ^18^O_DO_ values, down to + 17.3 ‰, and a much lower median value (+ 20.2 ‰) than the expected value for atmospheric equilibration (+ 23.6 ‰). On the other hand, most river and tributary samples exceeded this threshold (up to + 27.7 ‰), with a median of + 25.1 ‰. These generally higher values were most evident in December and October (Fig. 2).

**Fig. 3:**
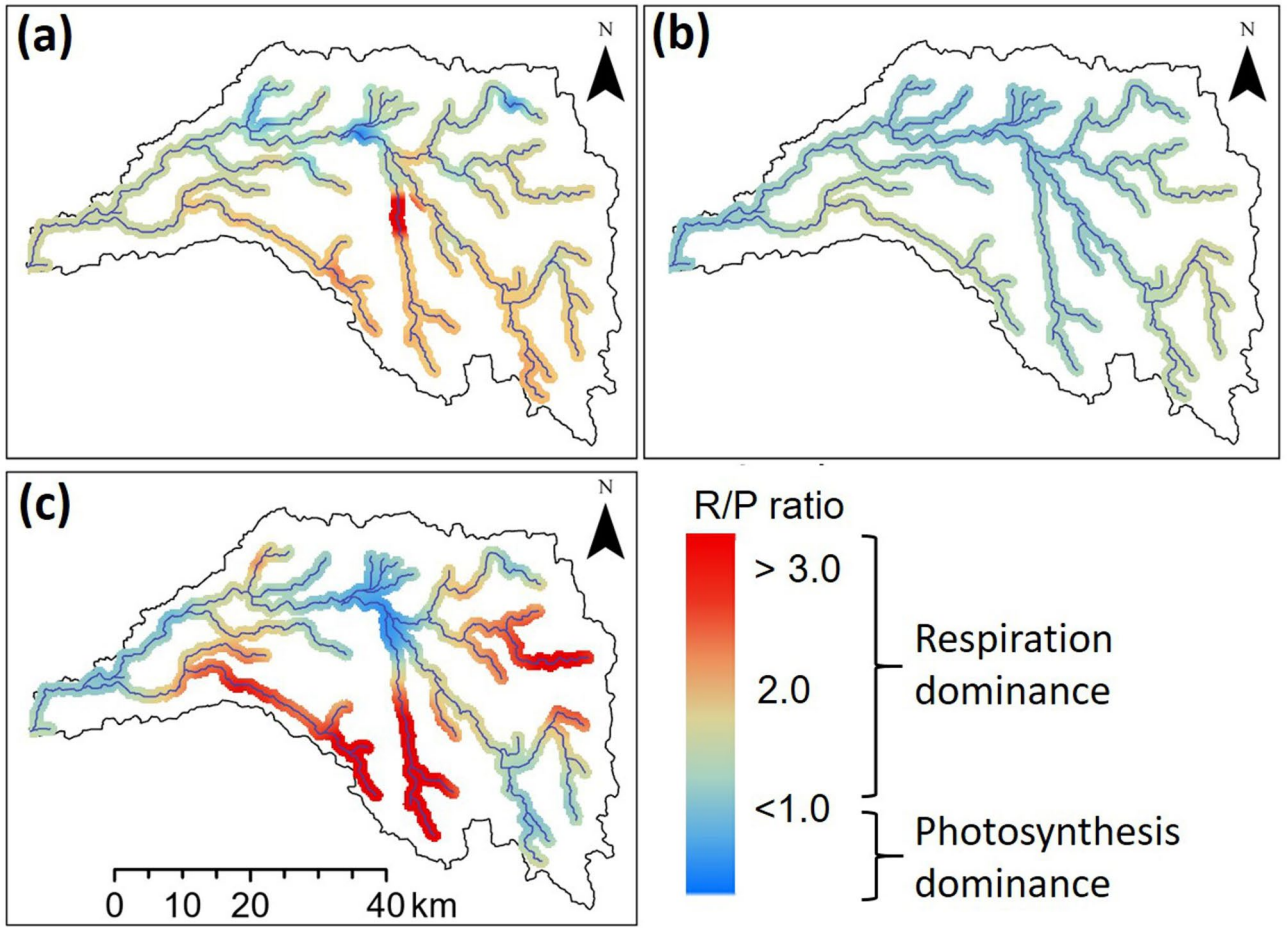
Spatial variations of R/P ratios of surface water in December (**a**), April (**b**), and October (**c**).

Calculated R/P ratios ranged from 0.5 to 4.5 (Table S1), with the highest ones in the southern part of the basin and the lowest ones in the reservoirs connected to the river. These spatial variations outlined the separation between P- and R-dominated river segments. Note that this separation was most evident in October (Fig. 3c). During this time, the southern and eastern headwaters showed R/P ratios > 3. In contrast, reservoirs and their downstream reaches showed lower R/P ratios near 1 (Fig. 3c). In December, similar trends were evident, though less pronounced than in October (Fig. 3a).

Measured DOC concentrations ranged from 0.12 to 1.28 mmol L^−1^ in the entire data set (Table S1). DOC concentrations showed clear seasonal variation, with a higher median of 0.65 mmol L^−1^ in October than in other seasons (*p* < 0.05). POC concentrations ranged from 0.01 to 0.40 mmol L^−1^ and showed clear seasonal variation in December, with a lower median of 0.06 mmol L^−1^ than in other seasons (*p* < 0.05). Calculated organic matter concentrations (sum of DOC and POC) ranged from 0.12 to 1.36 mml L^−1^ in the entire data set and showed a higher mean of 0.79 mmol L^−1^ in October than in other seasons (*p* < 0.05) (Table S1).

## Discussion

As expected by Henry’s law, the dataset shows a declining trend between DO concentrations and temperature. High median water temperatures in April (31 °C) were associated with a low median DO of 6.4 mg L^−1^. In contrast, lower median temperatures in December of 26 °C showed a higher median DO of 7.5 mg L^−1^. This relationship confirms that seasonal temperature changes are important for absolute DO levels. If temperature were the main factor affecting DO levels in the Deduru Oya River, it would be expected to reach 100% saturation level of 7.1 mg L^−1^ at the highest recorded temperature of 34 °C, as predicted by Henry’s Law (Eq. 4). Similarly, δ^18^O_DO_ values would be expected to remain in the range of + 24.6 ‰. However, the observed high variability in DO concentrations and δ^18^O_DO_ values suggests the presence of additional sinks and sources. This is also supported by δ^18^O_DO_ data indicating strong trends in photosynthesis and respiration.

Monthly rainfall in the Deduru Oya Basin varied in the order October > December > April, while monthly river discharge determined at the mouth ranged in the order December > October > April (Fig. 1e). Both processes seem to trigger DO concentration changes (Table S1). Therefore, fluctuations in DO in the Deduru Oya River could closely correlate with its discharge patterns. To further investigate this relationship, future studies should include discharge data, which are only sparsely available for the Deduru Oya River. In principle, an increase in discharge usually enhances turbulence, which favours O_2_ dissolution from the atmosphere (A). This input was further enhanced by monsoons, which cool the river.

Moreover, during all sampling campaigns, the headwater regions and reservoirs showed the highest DO concentrations. In headwater streams, elevated DO levels were associated with cooler temperatures and increased turbulence in steep terrain, both of which increased atmospheric input (A). Moreover, in reservoirs and downstream, elevated DO levels were likely controlled by P, which was enhanced by clearer, calmer waters. On the other hand, land use likely caused spatial DO trends mostly during April and October. During these times, land preparation for rice paddies increased connectivity to the river^53^. Exchange between rice paddies and the river enabled the addition of oxygen-depleted waters together with the addition of fresh organic material that became available for R. Repeated cycles of filling and draining water in rice paddy fields on the crop production stages have been shown to increase organic matter concentrations in adjacent rivers and streams, which in turn degrade water quality and lower DO levels^68–71^.

**Fig. 4:**
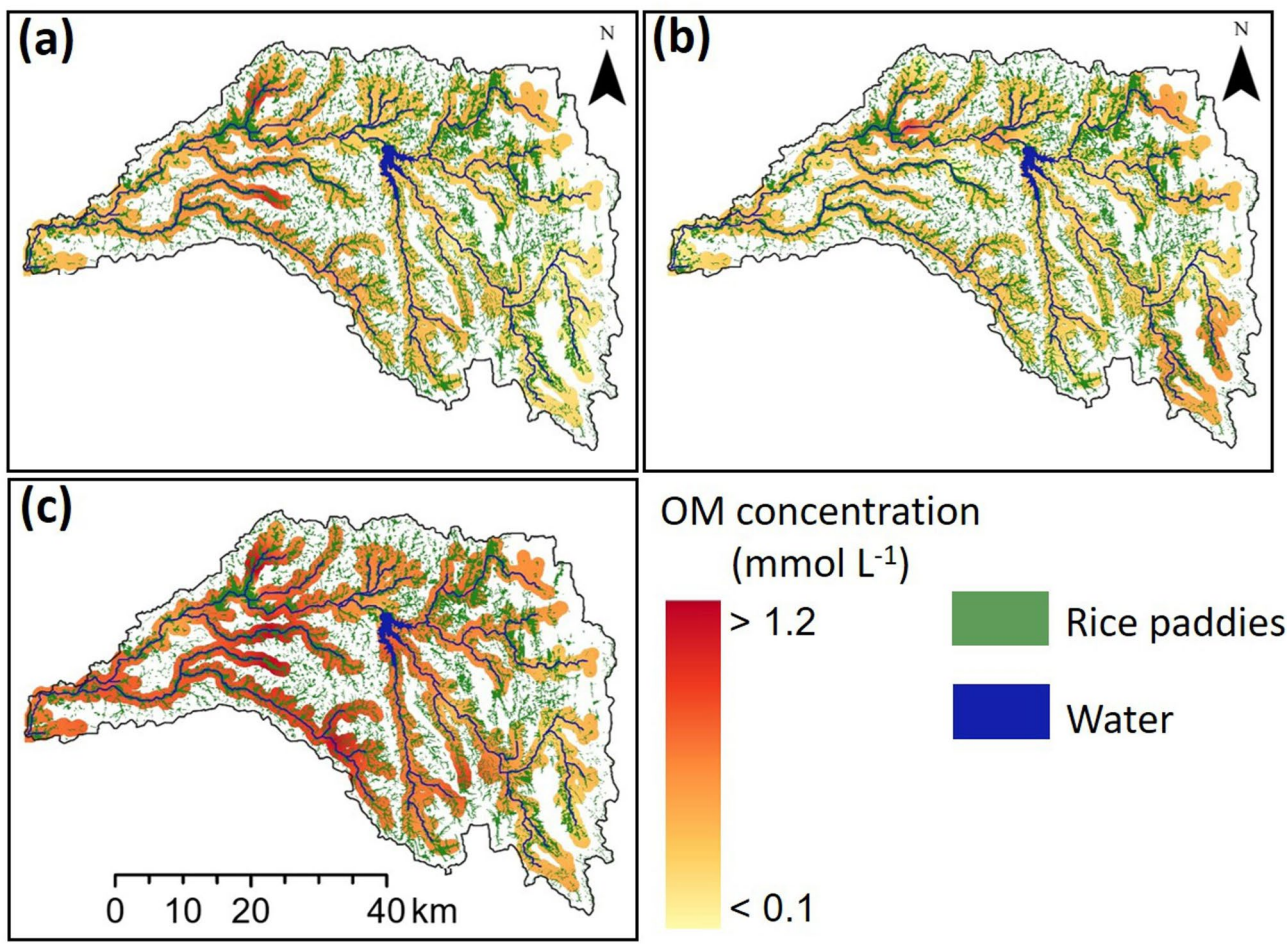
Spatial variations of organic matter (OM) concentrations of surface water in December (**a**), April (**b**), and October (**c**) with rice-paddy lands.

Figure 4 shows the spatial trends of organic matter (sum of DOC & POC) along the Deduru Oya River and its major streams together with rice paddy lands. The spatial trends of R/P in Fig. 3c and organic matter concentrations in Fig. 4c agree well with the above statement that increased organic matter content in the Deduru Oya River lowers DO levels through dominance of R.

Therefore, it is important to consider combinations of key drivers of DO. Its isotope variations help to scale metabolic processes with respect to the dissolution of atmospheric O_2_^72^. By concept, δ^18^O_DO_ values above the atmospheric equilibrium value of + 24.6 ‰ indicate R, while values below this threshold indicate P^73^. For background explanation, the atmospheric δ^18^O_O2_ value is constant at + 23.9 ‰, and its dissolution in water causes temperature-dependent isotope shifts of + 0.7 ‰ with little variation (± 0.1 ‰)^39,74^. R preferentially consumes ^16^O and thus shifts isotope ratios towards ^18^O-enriched values (i.e., more positive δ^18^O_DO_)^75^. P, on the other hand, splits the water molecule and imparts the liberated O_2_ into the DO pool with negligible fractionation^76,77^. Most water δ^18^O_H2O_ values in the Deduru Oya River are more enriched in ^16^O and range between 0.0 and − 6.8 ‰ (cf. supplementary text 1 and Table S1). Therefore, photosynthesis provides oxygen to the DO pool with an isotope value (δ^18^O_H2O_) that is enriched in ^16^O when compared to the atmospheric O_2_ value (δ^18^O_O2_)^39,78^. This results in lower δ^18^O_O2_ than for air_-_equilibrated water.

**Fig. 5:**
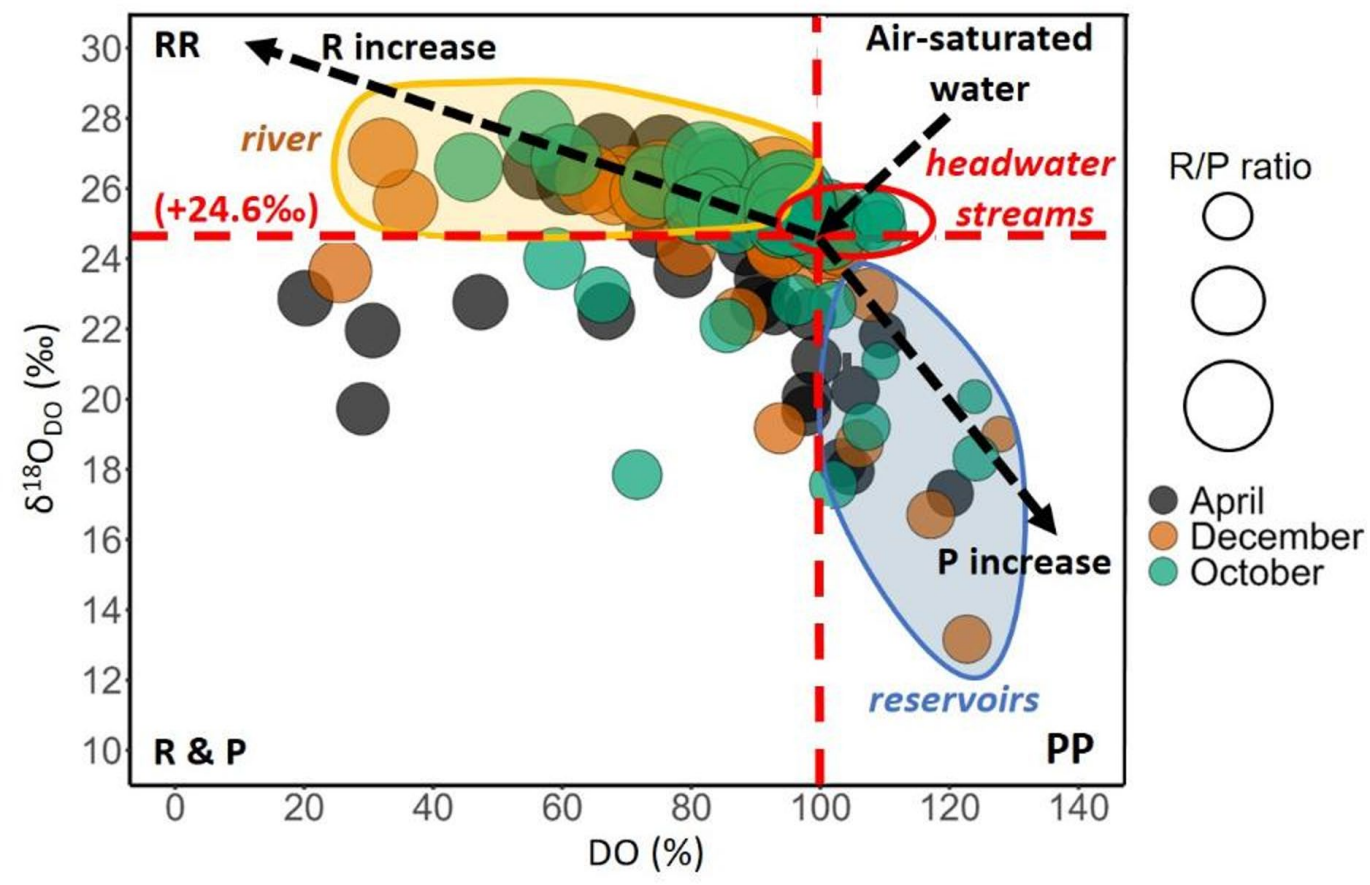
DO % versus δ^18^O_DO_ of surface water samples in the Deduru Oya River. Red dashed lines indicate saturation values with the atmosphere. Field PP is dominated by photosynthesis; R & P show mixed influences; and RR is dominated by respiration. The blue shaded area marks samples from the reservoirs and their nearby downstream river sections. The yellow-shaded area marks river sections that were hardly influenced by reservoirs and had increased loads of organic matter.

When plotting δ^18^O_DO_ versus DO %, the expected atmospheric equilibrium oxygen value of + 24.6 ± 0.4 ‰^39^ marks the boundary between photosynthesis and respiration on the y-axis of Fig. 5. A similar R-P separation also exists on the x-axis with DO % > 100 for increase of P and < 100 for increase of R.

Regardless of the season, most headwater samples scattered around the atmospheric equilibrium value (Fig. 5). These waters from the wet zone with rainfall > 200 cm yr^−1^ and steep gradients, undergo stronger turbulence leading to enhanced dissolution of atmospheric O_2_. Similar elevational patterns of DO associated with rainfall, runoff, and temperature variations are also observed in other tropical and sub-tropical headwater streams^79–81^. Further downstream, active P can shift δ^18^O_DO_ values down to + 17.3 ‰. This trend mostly occurs in reservoirs and downstream often leads to DO oversaturation (blue field in Fig. 5). Given favorable temperature and light conditions, algae can grow readily, leading to high photosynthetic production in the epilimnion^82–85^. Therefore, we assume that enhanced photosynthetic activity in the epilimnion of reservoirs shifted δ^18^O_DO_ away from atmospheric equilibrium toward ^16^O-enriched values (< + 24.6 ‰), with supersaturated DO levels.

Overall, the studied surface water samples exhibited positive correlations between δ^18^O_DO_ values and R/P ratios across varying intensities within each season (Fig. S2). This observation aligns with certain reservoir and near-reservoir water samples, which exhibit low DO % and more positive δ^18^O_DO_ values than those expected for atmospheric equilibration (Fig. 5). These samples demonstrated a trend shifting from the PP domain toward the respiration-photosynthesis mixed domain (R–P mixed field in Fig. 5), particularly during April and October (Fig. 5). Furthermore, most low DO % surface water samples within the R-P mixed domain correspond to high organic matter turnover rates, as reported by another study of this river. This study showed that organic matter accounts for up to 16% of the total carbon pool^86^.

Furthermore, during heavy monsoon runoff, soil, riparian vegetation, agricultural fertilisers, along with a large nutrient load, can become more easily transported to this river cascade system^87^. This is well demonstrated by the findings of Jayasiri et al.^87^. These authors reported high ammonium concentrations in the major paddy land preparation period in the Deduru Oya River basin, particularly in April^53,87^. Generally, Urea (CO(NH_2_)_2_) is one of the primary nitrogenous fertilizers applied to paddy lands, and this compound can easily transform into ammonium (NH_4_^+^), hydroxyl ion (OH^−^), and HCO_3_^−^^88^. In addition, 2–10% of these nitrogenous fertilizers can also be mixed with leaking waters from irrigation systems^89^. These nutrient- and organic-rich waters can stimulate microbial activity and lower DO levels through respiration and nitrification as follows:

aerobic decomposition (respiration)^90^:

$$Organic\;matter\left( {{\mathrm{CH}}_{2} {\mathrm{O}}} \right) + {\mathrm{O}}_{2} \to {\mathrm{CO}}_{2} + {\mathrm{H}}_{2} {\mathrm{O}} + energy$$

nitrification^91^:

$${\mathrm{NH}}_{4}^{ + } + 2{\mathrm{O}}_{2} \to {\mathrm{NO}}_{3}^{ - } + {\mathrm{H}}_{2} {\mathrm{O}} + 2{\mathrm{H}}^{ + } .$$

Notably, Jayasiri et al.^87^ reported clear seasonal variations in ammonium and nitrate concentrations in the Deduru Oya River during the wet and dry seasons. Consistent with these observations, the waters of the Deduru Oya River showed a strong influence of respiration (high R/P ratios). This finding could also imply the coexistence of DO depletion with OM decomposition and nitrification.

## Conclusions

Our study demonstrated strong seasonal and spatial variations in DO in the Deduru Oya River in Sri Lanka, which often reached hypoxic levels. This study is among the few that consider stable isotopes as additional tracers to concentration patterns in rivers. It allows quantification of R/P ratios under variable spatial and climatic conditions.

The river is affected by warm temperatures and monsoons, which increase the transport of organic matter via overland flow combined with rice farming and likely input of further untreated sewage. Further organic matter input via excessive rice farming, typical of many tropical regions, likely enhanced R, as demonstrated by typical δ^18^O_DO_ values more positive than those for atmospheric equilibration. These combinations of high temperatures and enhanced organic matter processing show the vulnerability of oxygen budgets in tropical rivers.

Coupling of DO concentrations and associated δ^18^O_DO_ helped to identify DO sources and sinks. DO source areas included the headwaters with atmospherically equilibrated cooler waters. Other sources were large reservoirs that connect to the river. Here, DO input was mainly by photosynthesis in epilimnion layers that were in turn favoured by enhanced nutrients and subsequent increased light penetration, as well as by stagnant flow.

Even though tropical rivers are particularly vulnerable to hypoxia, photosynthesis can counteract it. Ecosystem management strategies should therefore consider enhancing P levels, while reducing organic matter input would further support ecosystem restoration. Future studies should include diel observations of DO patterns and correlate them with chlorophyll, dissolved and particulate carbon concentrations, and their isotope ratios.

## Supplementary Information

Below is the link to the electronic supplementary material.

Supplementary Material 1

## Data Availability

Additional data related to this paper are available from the corresponding authors upon request.

## References

[1] Huang, W., Weng, N., Zhang, J. & Huo, S. Spatiotemporal patterns and driving factors of dissolved oxygen dynamics in a highly turbid subtropical macrotidal river estuary. *Mar. Pollut. Bull.* **222**, 118732 (2026). 40974797 10.1016/j.marpolbul.2025.118732

[2] Kulkarni, S. J. A review on research and studies on dissolved oxygen and its affecting parameters. (2016).

[3] Mainali, J. & Chang, H. Environmental and spatial factors affecting surface water quality in a Himalayan watershed, Central Nepal. *Environ. Sustain. Indic.* **9**, 100096 (2021).

[4] Piatka, D. R. et al. Dissolved oxygen isotope modelling refines metabolic state estimates of stream ecosystems with different land use background. *Sci. Rep.* **12**, 10204 (2022). 35715436 10.1038/s41598-022-13219-9 PMC9205993

[5] van Vliet, M. T. H. et al. Global river water quality under climate change and hydroclimatic extremes. *Nat. Rev. Earth Environ.* **4**, 687–702 (2023).

[6] Hutchings, A. M., de Vries, C. S., Hayes, N. R. & Orr, H. G. Temperature and dissolved oxygen trends in English estuaries over the past 30 years. *Estuar. Coast. Shelf Sci.* **306**, 108892 (2024).

[7] Null, S. E., Mouzon, N. R. & Elmore, L. R. Dissolved oxygen, stream temperature, and fish habitat response to environmental water purchases. *J. Environ. Manage.* **197**, 559–570 (2017). 28419978 10.1016/j.jenvman.2017.04.016

[8] Rajwa-Kuligiewicz, A., Bialik, R. J. & Rowiński, P. M. Dissolved oxygen and water temperature dynamics in lowland rivers over various timescales. *J. Hydrol. Hydromech.* **63**, 353–363 (2015).

[9] Li, L. et al. River water quality shaped by land–river connectivity in a changing climate. *Nat. Clim. Change* **14**, 225–237 (2024).

[10] Rajesh, M. & Rehana, S. Impact of climate change on river water temperature and dissolved oxygen: Indian riverine thermal regimes. *Sci. Rep.* **12**, 9222 (2022). 35655079 10.1038/s41598-022-12996-7 PMC9163182

[11] Guan, Q., Shi, K. & Pi, X. Sustained deoxygenation in global flowing waters under climate warming. *Sci. Adv.* **12**, eaef3132 (2026). 42139349 10.1126/sciadv.aef3132 PMC13178549

[12] Zhi, W., Klingler, C., Liu, J. & Li, L. Widespread deoxygenation in warming rivers. *Nat. Clim. Change* **13**, 1105–1113 (2023).

[13] Zhang, Q. et al. The effect of minimum temperatures on changes in river dissolved oxygen is more pronounced in the climate transition zone. *J. Environ. Manag.* **396**, 128043 (2025). 10.1016/j.jenvman.2025.128043 41274267

[14] Adelson, A. E. et al. Seasonal hypoxia and temperature inversions in a tropical bay. *Limnol. Oceanogr.* **67**, 2174–2189 (2022).

[15] Eriksen, T. E. et al. Effects of pollution-induced changes in oxygen conditions scaling up from individuals to ecosystems in a tropical river network. *Sci. Total Environ.* **814**, 151958 (2022). 34843774 10.1016/j.scitotenv.2021.151958

[16] Syvitski, J. P. M., Cohen, S., Kettner, A. J. & Brakenridge, G. R. How important and different are tropical rivers?—An overview. *Geomorphology* **227**, 5–17 (2014).

[17] Milliman, J. D. & Farnsworth, K. L. *River Discharge to the Coastal Ocean: A Global Synthesis* (Cambridge University Press, 2013).

[18] Huang, T.-H., Fu, Y.-H., Pan, P.-Y. & Chen, C.-T.A. Fluvial carbon fluxes in tropical rivers. *Curr. Opin. Environ. Sustain.* **4**, 162–169 (2012).

[19] Norouzi, S. et al. Dissolved organic matter quantity and quality response of tropical rainforest headwater rivers to the transition from dry to wet season. *Sci. Rep.* **14**, 3270 (2024). 38332222 10.1038/s41598-024-53362-z PMC10853192

[20] Sadhwani, K., Eldho, T. I. & Karmakar, S. Investigating the influence of future landuse and climate change on hydrological regime of a humid tropical river basin. *Environ. Earth Sci.* **82**, 210 (2023).

[21] Yun, M., Zhang, C., Wang, B., Huang, J. & Sun, J. The effects and mechanism of organic matter degradation in river sediment driven by humic-reducing bacteria. *J. Water Process Eng.* **67**, 106150 (2024).

[22] González, S. O., Almeida, C. A., Calderón, M., Mallea, M. A. & González, P. Assessment of the water self-purification capacity on a river affected by organic pollution: Application of chemometrics in spatial and temporal variations. *Environ. Sci. Pollut. Res.* **21**, 10583–10593 (2014). 10.1007/s11356-014-3098-y 24888622

[23] Atkins, M. L., Santos, I. R., Ruiz-Halpern, S. & Maher, D. T. Carbon dioxide dynamics driven by groundwater discharge in a coastal floodplain creek. *J. Hydrol.* **493**, 30–42 (2013).

[24] Pawson, R. R., Lord, D. R., Evans, M. G. & Allott, T. E. H. Fluvial organic carbon flux from an eroding peatland catchment, southern Pennines, UK. *Hydrol. Earth Syst. Sci.* **12**, 625–634 (2008).

[25] Sarkar, S., Verma, S., Begum, M. S., Park, J.-H. & Kumar, S. Sources, supply, and seasonality of total suspended matter and associated organic carbon and total nitrogen in three large Asian rivers—Ganges, Mekong, and Yellow. *Front. Earth Sci.*10.3389/feart.2023.1067744 (2023).

[26] Huang, C. & Zhang, C. Time-series remote sensing of rice paddy expansion in the Yellow River Delta: Towards sustainable ecological conservation in the context of water scarcity. *Remote Sens. Ecol. Conserv.* **9**, 454–468 (2023).

[27] Hutchins, M. G. et al. Intense summer floods may induce prolonged increases in benthic respiration rates of more than one year leading to low river dissolved oxygen. *J. Hydrol. X* **8**, 100056 (2020).

[28] Figueroa-Nieves, D., McDowell, W. H., Potter, J. D., Martínez, G. & Ortiz-Zayas, J. R. Effects of sewage effluents on water quality in tropical streams. *J. Environ. Qual.* **43**, 2053–2063 (2014). 25602222 10.2134/jeq2014.03.0139

[29] Mendivil-García, K. et al. Climate change impact assessment on a tropical river resilience using the Streeter-Phelps dissolved oxygen model. *Front. Environ. Sci.*10.3389/fenvs.2022.903046 (2022).

[30] Wang, F., Maberly, S. C., Wang, B. & Liang, X. Effects of dams on riverine biogeochemical cycling and ecology. *Inland Waters* **8**, 130–140 (2018).

[31] Nilsson, C., Reidy, C. A., Dynesius, M. & Revenga, C. Fragmentation and flow regulation of the world’s large river systems. *Science* **308**, 405–408 (2005). 15831757 10.1126/science.1107887

[32] March, J. G., Benstead, J. P., Pringle, C. M. & Scatena, F. N. Damming tropical island streams: Problems, solutions, and alternatives. *Bioscience* **53**, 1069–1078 (2003).

[33] Silva, E. I. L., Jennerjahn, T. C. & Ittekkot, V. Nutrient fluxes into coastal waters via Sri Lankan rivers: A comparison with other Asian rivers. *Int. J. Ecol. Environ. Sci.* **31**, 213–221 (2005).

[34] Lewis, A. S. L. et al. Reservoir drawdown highlights the emergent effects of water level change on reservoir physics, chemistry, and biology. *J. Geophys. Res. Biogeosci.* **129**, e2023JG007780 (2024).

[35] Shen, H., Ye, L., Cai, Q. & Tan, L. Longitudinal variations in physiochemical conditions and their consequent effect on phytoplankton functional diversity within a subtropical system of cascade reservoirs. *Front. Ecol. Evol.* **10**, 914623 (2022).

[36] Zhong, M. et al. Modeling spatial patterns of dissolved oxygen and the impact mechanisms in a cascade river. *Front. Environ. Sci.*10.3389/fenvs.2021.781646 (2021).

[37] Barth, J. A. C., Tait, A. & Bolshaw, M. Automated analyses of ^18^O/^16^O ratios in dissolved oxygen from 12-mL water samples. *Limnol. Oceanogr. Methods* **2**, 35–41 (2004).

[38] Dordoni, M., Seewald, M., Rinke, K., Schmidmeier, J. & Barth, J. A. C. Novel evaluations of sources and sinks of dissolved oxygen via stable isotopes in lentic water bodies. *Sci. Total Environ.* **838**, 156541 (2022). 35679920 10.1016/j.scitotenv.2022.156541

[39] Mader, M., Schmidt, C., Van Geldern, R. & Barth, J. A. C. Dissolved oxygen in water and its stable isotope effects: A review. *Chem. Geol.* **473**, 10–21 (2017).

[40] Maier, J., Visser, A.-N., Schubert, C. M., Wander, S. T. & Barth, J. A. C. Hydrodynamic and primary production effects on seasonal DO variability in the Danube River. *Biogeosciences* **22**, 5123–5137 (2025).

[41] Hridoy, M. A. A. M. et al. Operational agricultural water management in river-based aquaculture: A machine-learning approach to predict dissolved oxygen in the Halda River, Bangladesh. *Aquac. Eng.*10.1016/j.aquaeng.2026.102693 (2026).

[42] Rixen, T. et al. Dissolved oxygen and its response to eutrophication in a tropical black water river. *J. Environ. Manag.* **91**, 1730–1737 (2010). 10.1016/j.jenvman.2010.03.009 20435403

[43] Xu, Y. et al. How anthropogenic factors influence the dissolved oxygen in surface water over three decades in eastern China?. *J. Environ. Manag.* **326**, 116828 (2023). 10.1016/j.jenvman.2022.116828 36436243

[44] Dutton, C. L., Subalusky, A. L., Hamilton, S. K., Rosi, E. J. & Post, D. M. Organic matter loading by hippopotami causes subsidy overload resulting in downstream hypoxia and fish kills. *Nat. Commun.* **9**, 1951 (2018). 29769538 10.1038/s41467-018-04391-6 PMC5956076

[45] Rollnic, M. et al. Environmental background in Amazonian rivers near the industrial pole, northern Brazil. *Sci. Rep.* **16**, 15899 (2026). 41933066 10.1038/s41598-026-44852-3 PMC13194907

[46] Mapa, R. B. (ed.) *The Soils of Sri Lanka* 119–124 (Springer, 2020). 10.1007/978-3-030-44144-9.

[47] Punyawardena, B. V. R. Climate. In *The Soils of Sri Lanka* (ed. Mapa, R. B.) 13–22 (Springer, 2020). 10.1007/978-3-030-44144-9_2.

[48] Jayawardena, I. M. S. P., Darshika, D. W. T. T. & Herath, H. M. R. C. Recent trends in climate extreme indices over Sri Lanka. *Am. J. Clim. Change* **07**, 586–599 (2018).

[49] Perera, C. et al. Designing for the past in a nonstationary climate: Evidence from Cyclone Ditwah’s extreme rainfall in Sri Lanka. *Hydrology* **13**, 47 (2026).

[50] Somasundaram, D. et al. Spatial and temporal changes in surface water area of Sri Lanka over a 30-year period. *Remote Sens.* **12**, 3701 (2020).

[51] Jayasena, H. A., Chandrajith, R. & Gangadara, K. R. Water management in ancient tank cascade systems (TCS) in Sri Lanka: Evidence for systematic tank distribution. In *Water Management in Ancient Tank Cascade Systems (TCS) in Sri Lanka: Evidence for Systematic Tank Distribution 29–34 H.A.H. Jayasena, Rohana Chandrajith and K.R. Gangadhara PDF (size 587 kb)*, Vol. 14, 29–34 (2011).

[52] Jayasena, H. A. H., Chandrajith, R. & Dissanayake, C. B. Spatial variation of isotope composition in precipitation in a tropical environment: A case study from the Deduru Oya river basin, Sri Lanka. *Hydrol. Process.* **22**, 4565–4570 (2008).

[53] Weerakoon, S. B., Sampath, S. & Herath, S. Integrated water resources analysis of the Deduru Oya Left Bank considering traditional and modern systems.

[54] Wanninayake, W. M. S. B. et al. Integrated approach to addressing flood risk in Sri Lanka’s Deduru Oya Basin: Implications for water-food-land-energy nexus. Preprint at 10.20944/preprints202404.1906.v1 (2024).

[55] Chandrasiri, S., Galagedara, L. & Mowjood, M. Impacts of rainfall variability on paddy production: A case from Bayawa minor irrigation tank in Sri Lanka. *Paddy Water Environ.* **18**, 443–454 (2020).

[56] Wassenaar, L. I. & Koehler, G. An on-line technique for the determination of the δ^18^O and δ^17^O of gaseous and dissolved oxygen. *Anal. Chem.* **71**, 4965–4968 (1999). 21662841 10.1021/ac9903961

[57] van Geldern, R. & Barth, J. A. C. Optimization of instrument setup and post-run corrections for oxygen and hydrogen stable isotope measurements of water by isotope ratio infrared spectroscopy (IRIS). *Limnol. Oceanogr. Methods* **10**, 1024–1036 (2012).

[58] Baertschi, P. Absolute ^18^O content of standard mean ocean water. *Earth Planet. Sci. Lett.* **31**, 341–344 (1976).

[59] Quay, P. D. et al. The ^18^O:^16^O of dissolved oxygen in rivers and lakes in the Amazon Basin: Determining the ratio of respiration to photosynthesis rates in freshwaters. *Limnol. Oceanogr.* **40**, 718–729 (1995).

[60] Russ, M. E., Ostrom, N. E., Gandhi, H., Ostrom, P. H. & Urban, N. R. Temporal and spatial variations in R:P ratios in Lake Superior, an oligotrophic freshwater environment. *J. Geophys. Res. Oceans* **109** (2004).

[61] Stevens, C. L. R., Schultz, D., Van Baalen, C. & Parker, P. L. Oxygen isotope fractionation during photosynthesis in a blue-green and a green alga 1 2. *Plant Physiol.* **56**, 126–129 (1975). 16659241 10.1104/pp.56.1.126 PMC541311

[62] Knox, M., Quay, P. D. & Wilbur, D. Kinetic isotopic fractionation during air-water gas transfer of O[sub 2], N[sub 2], CH[sub 4], and H[sub 2]. *J. Geophys. Res. (U. S.)* **97**, C12 (1992).

[63] Benson, B. B. & Krause, D. Jr. The concentration and isotopic fractionation of oxygen dissolved in freshwater and seawater in equilibrium with the atmosphere. *Limnol. Oceanogr.* **29**, 620–632 (1984).

[64] St-Jean, G. Automated quantitative and isotopic (13C) analysis of dissolved inorganic carbon and dissolved organic carbon in continuous-flow using a total organic carbon analyser. *Rapid Commun. Mass Spectrom.* **17**, 419–428 (2003). 12590390 10.1002/rcm.926

[65] van Geldern, R. et al. Stable carbon isotope analysis of dissolved inorganic carbon (DIC) and dissolved organic carbon (DOC) in natural waters—Results from a worldwide proficiency test. *Rapid Commun. Mass Spectrom.* **27**, 2099–2107 (2013). 23943331 10.1002/rcm.6665

[66] Saeed, O. et al. Assessing surface water quality in Hungary’s Danube basin using geochemical modeling, multivariate analysis, irrigation indices, and Monte Carlo simulation. *Sci. Rep.* **14**, 18639 (2024). 39128943 10.1038/s41598-024-69312-8 PMC11317494

[67] Shehata, M. et al. Integrated management of groundwater quantity, physicochemical properties, and microbial quality in West Nile delta using a new MATLAB code and geographic information system mapping. *Sci. Rep.* **14**, 7762 (2024). 38565529 10.1038/s41598-024-57036-8 PMC10987591

[68] Khan, M. N. N., Zainol, M. R. R. M. A., Yusop, M. F. M., Ahmad, M. A. & Latiff, M. F. P. M. Quantifying the impact: Statistical analysis of weather and paddy activities on paddy effluent quality. *Kuwait J. Sci.* **53**, 100471 (2026).

[69] Yamaoka, L. *Water and Agriculture Sustainability, Markets and Policies: Sustainability, Markets and Policies* (OECD Publishing, 2006).

[70] Gustinasari, K., Hermana, J. & Pandebesie, E. S. Water bodies quality along paddy field in Karang Ploso Sub District, Malang City, Indonesia. *E3S Web Conf.* **125**, 04007 (2019).

[71] Loi, J. X. et al. Water quality assessment and pollution threat to safe water supply for three river basins in Malaysia. *Sci. Total Environ.* **832**, 155067 (2022). 35395310 10.1016/j.scitotenv.2022.155067

[72] Dugan, H. A. et al. Consequences of gas flux model choice on the interpretation of metabolic balance across 15 lakes. *Inland Waters* **6**, 581–592 (2016).

[73] Bass, A. M., Waldron, S., Preston, T., Adams, C. E. & Drummond, J. Temporal and spatial heterogeneity in lacustrine δ13CDIC and δ18ODO signatures in a large mid-latitude temperate lake. *J. Limnol.* **69**, 341 (2010).

[74] Barkan, E. & Luz, B. Diffusivity fractionations of HO/HO and HO/HO in air and their implications for isotope hydrology. *Rapid Commun. Mass Spectrom.* **21**, 2999–3005 (2007). 17705344 10.1002/rcm.3180

[75] Wassenaar, L. I., Crespel, A., Barth, J. A. C., Koeck, B. & Závorka, L. Non-invasive determination of critical dissolved oxygen thresholds for stress physiology in fish using triple-oxygen stable isotopes and aquatic respirometry. *Isot. Environ. Health Stud.* **60**, 365–379 (2024). 10.1080/10256016.2024.2366470 38949394

[76] Mader, M. et al. River recharge versus O2 supply from the unsaturated zone in shallow riparian groundwater: A case study from the Selke River (Germany). *Sci. Total Environ.* **634**, 374–381 (2018). 29627561 10.1016/j.scitotenv.2018.03.230

[77] Eisenstadt, D., Barkan, E., Luz, B. & Kaplan, A. Enrichment of oxygen heavy isotopes during photosynthesis in phytoplankton. *Photosynth. Res.* **103**, 97–103 (2010). 20054712 10.1007/s11120-009-9518-z

[78] Lane, G. A. & Dole, M. Fractionation of oxygen isotopes during respiration. *Science* **123**, 574–576 (1956). 13311503 10.1126/science.123.3197.574

[79] Wang, L. et al. Elevational patterns of tropical stream insect communities are associated with annual mean temperature, aluminium and dissolved oxygen concentrations. *Freshw. Biol.* **71**, e70233 (2026).

[80] Carter, A. M., DelVecchia, A. G. & Bernhardt, E. S. Patterns and drivers of dissolved gas concentrations and fluxes along a low gradient stream. *JGR Biogeosci.* **127**, e2022JG007048 (2022).

[81] Canadell, M. B., Gómez‐Gener, L., Clémençon, M., Lane, S. N. & Battin, T. J. Daily entropy of dissolved oxygen reveals different energetic regimes and drivers among high‐mountain stream types. *Limnol. Oceanogr.* **66**, 1594–1610 (2021).

[82] Barjau-Aguilar, M. et al. Diversity and structure of the prokaryotic community in tropical monomictic reservoir. *Microb. Ecol.* **88**, 12 (2025). 40072582 10.1007/s00248-025-02508-1 PMC11903632

[83] Ma, B., Dong, F., Peng, W., Liu, X. & Huang, A. Dynamics of oxygen evolution in a thermally stratified reservoir under climate warming. *Sci. Rep.* **15**, 40419 (2025). 41253844 10.1038/s41598-025-13432-2 PMC12627531

[84] Yu, Z., Yang, J., Amalfitano, S., Yu, X. & Liu, L. Effects of water stratification and mixing on microbial community structure in a subtropical deep reservoir. *Sci. Rep.* **4**, 5821 (2014). 25059241 10.1038/srep05821 PMC5376048

[85] Zhong, Y. et al. The spatiotemporal variations in microalgae communities in vertical waters of a subtropical reservoir. *J. Environ. Manag.* **317**, 115379 (2022). 10.1016/j.jenvman.2022.115379 35751236

[86] Senarathne, S., van Geldern, R., Chandrajith, R. & Barth, J. A. C. Unexpected contributions by carbonates and organic matter in a silicate-dominated tropical catchment: An isotope approach. *Sci. Total Environ.* **948**, 174651 (2024). 38992376 10.1016/j.scitotenv.2024.174651

[87] Jayasiri, M. M. J. G. C. N. et al. Spatio-temporal analysis of water quality for pesticides and other agricultural pollutants in *Deduru Oya* river basin of Sri Lanka. *J. Clean. Prod.* **330**, 129897 (2022).

[88] Kim, G. W., Jeong, S. T., Kim, G. Y., Kim, P. J. & Kim, S. Y. Evaluation of carbon dioxide emission factor from urea during rice cropping season: A case study in Korean paddy soil. *Atmos. Environ.* **139**, 139–146 (2016).

[89] Gunatilake, S. N and P variation in groundwater in wet zone and dry zone in Sri Lanka due to fertilization of paddy crop. *Int. J. Sci. Res. Publ.* **6**, 1–2250 (2016).

[90] Shang, H. A generic hierarchical model of organic matter degradation and preservation in aquatic systems. *Commun. Earth Environ.* **4**, 16 (2023).

[91] Edwards, T. M. et al. Ammonia and aquatic ecosystems—A review of global sources, biogeochemical cycling, and effects on fish. *Sci. Total Environ.* **907**, 167911 (2024). 37871823 10.1016/j.scitotenv.2023.167911

