# Supplementary material for: Tropical rivers face severe oxygen stress caused by heat and organic matter: new evidence by stable isotopes
Source: Sci Rep. 2026 Aug 10;16:24717. doi: 10.1038/s41598-026-65862-1 (PMC13458584; doi:10.1038/s41598-026-65862-1)
Supplement: Supplementary file 1 [file 41598_2026_65862_MOESM1_ESM.docx]

Tropical rivers face severe oxygen-stress caused by heat and organic matter: new evidence by stable isotopes

Sachintha Senarathne^1*^, Robert van Geldern^1^, Rohana Chandrajith^1,2^, Johannes A. C. Barth^1^

^1^Friedrich-Alexander-Universität Erlangen-Nürnberg (FAU), Department Geographie und Geowissenschaften, 91054, Erlangen, GeoZentrum Nordbayern, Schlossgarten 5, Germany.

^2^Department of Geology, Faculty of Science, University of Peradeniya, Peradeniya, Sri Lanka.

*Corresponding author: Sachintha Senarathne ([sachintha.senarathne@fau.de](mailto:sachintha.senarathne@fau.de))

**Text S1. Variation of stable water isotope (δ^18^O_H2O_)**

Water isotope values (δ^18^O_H2O_) ranged from -6.8 ‰ to +0.0 ‰ (Table S1). Highest δ^18^O_H2O_ values occurred in April and ranged from -4.3 to +0.0 ‰, while in December they ranged from -6.8 to -2.0 ‰ (Table S1). δ^18^O_H2O_ values in April significantly differed from those in December (p < 0.05). Moreover, pronounced spatial variation in δ^18^O_H2O_ occurred in October with values that range from -6.3 to -0.6 (Table S1). Headwater regions showed depleted δ^18^O_H2O_ when compared to downstream sections (Figure S1).

**Figure S1.** (a) Overview of Sri Lanka (left) and land use types in the Deduru Oya Basin with sampling points. Subfigures (b-d) show spatial variations of δ^18^O_H2O_ in December (b), April (c), and October (d).

**Text S2. Temperature dependence of Henry’s law constants and solubility of DO in freshwaters**

Henry's Law states that the concentration of a species in the aqueous phase is directly proportional to the pressure of that species in the gas phase under equilibrium conditions^92^. Various formulas in the literature describe the temperature dependence of gas solubility in water. A common approach to express the Henry’s law is as follows^1^:

$$K_{H} =\frac{C}{P}$$

Here, K_H_ is the Henry's solubility constant (mol m^-3^ Pa^-1^), C is the concentration (in mol m^-3^) of the dissolved gas and P is the partial pressure (in Pa) of the gas above the liquid phase under equilibrium conditions. For more information regarding the Henry’s law constants and related temperature dependence of gas solubility formulas can be found in Sander^93^. In general, oxygen solubility increases with lower water temperatures and higher atmospheric pressures, and decreases under the opposite conditions.

**Text S3. Additional information for data download and figure generation**

**Figures 1 to 4:** Maps were generated using ArcGIS Pro version 3.1 (https://www.esri.com) with raster data obtained from the United States Geological Survey (USGS) Earth Explorer (United States Geological Survey, 2024) via https://earthexplorer.usgs.gov. **Figure 1:** Land use data were sourced from the Survey Department of Sri Lanka (2022) via <https://survey.gov.lk>. **Figure 5:** This figure was created in RStudio 2025 (version 4.5.0) using the ggplot2 package (<https://r-graph-gallery.com>).

**Table S1.** Measured values and corresponding summary statistics of surface water in the Deduru Oya River

| **Sample** | **Coordinates** | | **Type** | **Temp.** | **DO** | **DO** | **δ^18^O_DO_** | **δ^18^O_H2O_** | **R/P** | **DOC** | **POC** | **OM** |
| --- | --- | --- | --- | --- | --- | --- | --- | --- | --- | --- | --- | --- |
| **NO.** | **N** | **E** |  | **(°C)** | **(mg L^-1^)** | **(%)** | **(‰)** | |  | **(mmol L^-1^)** | | |
| **December-2022** | | |  |  |  |  |  |  |  |  |  |  |
| 1 | 7.425 | 80.4598 | River | 26 | 7.9 | 102 | 24.9 | -5.2 | 3.3 | 0.22 | 0.088 | 0.3 |
| 2 | 7.4552 | 80.5248 | River | 25 | 7.9 | 99 | 24.9 | -5.3 | 2.3 | 0.17 | 0.023 | 0.2 |
| 3 | 7.5621 | 80.4973 | River | 25 | 8.2 | 103 | 24.4 | -5.4 | 1.3 | 0.2 | 0.030 | 0.2 |
| 4 | 7.5906 | 80.3665 | River | 26 | 7.9 | 102 | 24.3 | -5.9 | 1.4 | 0.20 | 0.036 | 0.2 |
| 5 | 7.6542 | 80.5041 | River | 26 | 7.3 | 93 | 24.3 | -5.2 | 1.6 | 0.22 | 0.058 | 0.3 |
| 6 | 7.6781 | 80.3701 | River | 26 | 7.9 | 100 | 23.9 | -3.7 | 1.2 | 0.34 | 0.061 | 0.4 |
| 7 | 7.7714 | 80.4456 | Reservoir | 28 | 9.7 | 128 | 19.0 | -3.4 | 0.5 | 0.43 | 0.106 | 0.5 |
| 8 | 7.741 | 80.3801 | River | 26 | 6.3 | 79 | 24.4 | -3.0 | 1.5 | 0.50 | 0.085 | 0.6 |
| 9 | 7.6996 | 80.3072 | Reservoir | 26 | 6.4 | 81 | 25.9 | -5.0 | 2.3 | 0.38 | 0.058 | 0.4 |
| 10 | 7.4304 | 80.333 | River | 27 | 2.5 | 32 | 27.0 | -3.7 | 2.0 | 0.46 | 0.083 | 0.5 |
| 11 | 7.4982 | 80.3842 | River | 26 | 5.7 | 73 | 25.9 | -4.3 | 2.0 | 0.51 | 0.082 | 0.6 |
| 12 | 7.547 | 80.3068 | River | 26 | 7.8 | 99 | 25.0 | -4.5 | 2.9 | 0.40 | 0.055 | 0.5 |
| 13 | 7.6266 | 80.2935 | River | 25 | 7.5 | 93 | 25.8 | -4.7 | 4.5 | 0.41 | 0.072 | 0.5 |
| 14 | 7.6705 | 80.2877 | Reservoir | 28 | 6.8 | 87 | 22.4 | -6.8 | 1.3 | 0.41 | 0.045 | 0.5 |
| 15 | 7.7179 | 80.274 | Reservoir | 27 | 8.0 | 108 | 23.0 | -5.6 | 1.0 | 0.33 | 0.032 | 0.4 |
| 16 | 7.7314 | 80.2427 | Reservoir | 26 | 8.3 | 108 | 24.9 | -5.3 | 1.4 | 0.34 | 0.059 | 0.4 |
| 17 | 7.8301 | 80.1398 | Reservoir | 27 | 2.0 | 26 | 23.6 | -5.3 | 1.6 | 0.49 | 0.031 | 0.5 |
| 18 | 7.7778 | 80.1066 | River | 31 | 8.6 | 117 | 16.7 | -2.0 | 1.0 | 0.99 | 0.173 | 1.2 |
| 19 | 7.7468 | 80.1035 | River | 29 | 9.3 | 123 | 13.2 | -4.9 | 1.0 | 0.49 | 0.026 | 0.5 |
| 20 | 7.747 | 80.1195 | Reservoir | 27 | 8.2 | 106 | 18.7 | -4.9 | 1.0 | 0.41 | 0.073 | 0.5 |
| 21 | 7.6933 | 80.1834 | River | 27 | 7.3 | 94 | 19.2 | -5.6 | 1.1 | 0.39 | 0.023 | 0.4 |
| 22 | 7.6954 | 80.1252 | River | 28 | 5.2 | 68 | 25.9 | -4.0 | 2.0 | 0.64 | 0.084 | 0.7 |
| 23 | 7.6977 | 79.9985 | River | 25 | 7.8 | 95 | 24.6 | -5.2 | 1.7 | 0.41 | 0.070 | 0.5 |
| 24 | 7.6495 | 80.1255 | River | 28 | 2.8 | 36 | 25.6 | -2.7 | 1.7 | 1.07 | 0.087 | 1.2 |
| 25 | 7.5494 | 80.2111 | River | 26 | 6.6 | 83 | 26.2 | -3.8 | 2.6 | 0.51 | 0.122 | 0.6 |
| 26 | 7.5755 | 80.2069 | River | 26 | 5.1 | 64 | 26.3 | -3.6 | 2.0 | 0.54 | 0.090 | 0.6 |
| 27 | 7.5557 | 80.192 | River | 26 | 6.9 | 87 | 25.6 | -3.9 | 2.2 | 0.52 | 0.090 | 0.6 |
| 28 | 7.615 | 80.0839 | River | 26 | 6.0 | 75 | 26.3 | -3.9 | 2.3 | 0.52 | 0.067 | 0.6 |
| 29 | 7.6378 | 80.0241 | River | 27 | 5.5 | 70 | 26.2 | -4.2 | 2.1 | 0.66 | 0.051 | 0.7 |
| 30 | 7.6192 | 79.9066 | River | 25 | 7.6 | 93 | 24.4 | -5.0 | 1.6 | 0.5 | 0.048 | 0.5 |
| 31 | 7.6041 | 79.8125 | Reservoir | 25 | 7.5 | 92 | 24.8 | -5.0 | 1.8 | 0.39 | 0.054 | 0.4 |
|  |  |  |  |  |  |  |  |  |  |  |  |  |
| Minimum | | |  | 25 | 2.0 | 26 | 13.2 | -6.8 | 0.5 | 0.16 | 0.02 | 0.19 |
| Maximum | | |  | 31 | 9.7 | 128 | 27.0 | -2.0 | 4.5 | 1.07 | 0.17 | 1.16 |
| Mean | | |  | 27 | 6.9 | 88 | 23.8 | -4.5 | 1.8 | 0.45 | 0.07 | 0.52 |
| Median | | |  | 26 | 7.5 | 93 | 24.8 | -4.9 | 1.7 | 0.41 | 0.06 | 0.49 |
| Standard deviation | | |  | 1 | 1.8 | 24 | 3.2 | 1.0 | 0.8 | 0.20 | 0.03 | 0.22 |
|  |  |  |  |  |  |  |  |  |  |  |  |  |
| **April-2023** | |  |  |  |  |  |  |  |  |  |  |  |
| 1 | 7.425 | 80.4598 | River | 27 | 7.6 | 98 | 24.6 | -4.3 | 1.7 | 0.42 | 0.226 | 0.6 |
| 2 | 7.4552 | 80.5248 | River | 27 | 7.5 | 97 | 24.7 | -3.7 | 1.7 | 0.43 | 0.344 | 0.8 |
| 3 | 7.5621 | 80.4973 | River | 28 | 7.3 | 96 | 25.2 | -3.2 | 2.4 | 0.4 | 0.251 | 0.6 |
| 4 | 7.5906 | 80.3665 | River | 33 | 7.6 | 110 | 21.8 | -2.3 | 0.8 | 0.31 | 0.067 | 0.4 |
| 5 | 7.6542 | 80.5041 | River | 30 | 6.6 | 90 | 25.2 | -4.3 | 2.0 | 0.38 | 0.119 | 0.5 |
| 6 | 7.6781 | 80.3701 | River | 32 | 6.4 | 90 | 22.7 | -2.1 | 1.2 | 0.27 | 0.044 | 0.3 |
| 7 | 7.7714 | 80.4456 | Reservoir | 33 | 7.0 | 99 | 21.1 | -2.3 | 1.0 | 0.56 | 0.113 | 0.7 |
| 8 | 7.741 | 80.3801 | River | 30 | 4.1 | 56 | 26.6 | -1.0 | 1.8 | 0.30 | 0.089 | 0.4 |
| 9 | 7.6996 | 80.3072 | Reservoir | 33 | 7.4 | 105 | 18.0 | -2.8 | 1.0 | 0.29 | 0.045 | 0.3 |
| 10 | 7.4304 | 80.333 | River | 31 | 3.4 | 47 | 22.8 | -1.0 | 1.3 | 0.24 | 0.029 | 0.3 |
| 11 | 7.4982 | 80.3842 | River | 33 | 6.4 | 91 | 23.4 | -1.3 | 1.2 | 0.36 | 0.065 | 0.4 |
| 12 | 7.547 | 80.3068 | River | 31 | 6.4 | 89 | 24.3 | -0.7 | 1.4 | 0.34 | 0.100 | 0.4 |
| 13 | 7.6266 | 80.2935 | River | 31 | 4.4 | 61 | 26.2 | -2.0 | 1.8 | 0.34 | 0.071 | 0.4 |
| 14 | 7.6705 | 80.2877 | Reservoir | 32 | 7.0 | 98 | 19.7 | -3.0 | 1.0 | 0.37 | 0.067 | 0.4 |
| 15 | 7.7179 | 80.274 | Reservoir | 31 | 5.8 | 79 | 23.7 | -2.7 | 1.4 | 0.25 | 0.067 | 0.3 |
| 16 | 7.7314 | 80.2427 | Reservoir | 31 | 7.6 | 103 | 18.2 | -2.9 | 1.0 | 0.47 | 0.163 | 0.6 |
| 17 | 7.8301 | 80.1398 | Reservoir | 30 | 2.1 | 29 | 19.7 | -2.4 | 1.2 | 0.19 | 0.036 | 0.2 |
| 18 | 7.7778 | 80.1066 | River | 30 | 5.9 | 80 | 25.6 | -0.3 | 1.7 | 0.42 | 0.133 | 0.6 |
| 19 | 7.7468 | 80.1035 | River | 33 | 6.9 | 98 | 20.1 | -2.7 | 1.0 | 0.51 | 0.161 | 0.7 |
| 20 | 7.747 | 80.1195 | Reservoir | 33 | 8.4 | 120 | 17.3 | -2.8 | 0.9 | 0.80 | 0.173 | 1.0 |
| 21 | 7.6933 | 80.1834 | River | 31 | 4.9 | 67 | 22.5 | -3.5 | 1.3 | 0.12 | 0.007 | 0.1 |
| 22 | 7.6954 | 80.1252 | River | 30 | 5.5 | 74 | 24.9 | -0.7 | 1.5 | 0.31 | 0.096 | 0.4 |
| 23 | 7.6977 | 79.9985 | River | 31 | 6.8 | 93 | 22.6 | -1.6 | 1.1 | 0.28 | 0.074 | 0.4 |
| 24 | 7.6495 | 80.1255 | River | 29 | 2.4 | 31 | 22.0 | -1.4 | 1.3 | 0.38 | 0.123 | 0.5 |
| 25 | 7.5494 | 80.2111 | River | 30 | 5.0 | 67 | 27.1 | 0.0 | 2.0 | 0.29 | 0.135 | 0.4 |
| 26 | 7.5755 | 80.2069 | River | 29 | 1.5 | 20 | 22.9 | -0.7 | 1.3 | 0.40 | 0.118 | 0.5 |
| 27 | 7.5557 | 80.192 | River | 30 | 5.9 | 79 | 26.1 | 0.0 | 1.9 | 0.39 | 0.174 | 0.6 |
| 28 | 7.615 | 80.0839 | River | 30 | 6.2 | 84 | 26.1 | -0.5 | 2.1 | 0.38 | 0.114 | 0.5 |
| 29 | 7.6378 | 80.0241 | River | 30 | 5.6 | 76 | 27.0 | -0.6 | 2.4 | 0.27 | 0.116 | 0.4 |
| 30 | 7.6192 | 79.9066 | River | 32 | 7.1 | 98 | 22.4 | -2.1 | 1.1 | 0.2 | 0.042 | 0.3 |
| 31 | 7.6041 | 79.8125 | Reservoir | 34 | 7.4 | 105 | 20.2 | -1.9 | 1.0 | 0.17 | 0.048 | 0.2 |
|  |  |  |  |  |  |  |  |  |  |  |  |  |
| Minimum | | | | 27 | 1.5 | 20 | 17.3 | -4.3 | 0.8 | 0.12 | 0.01 | 0.12 |
| Maximum | | | | 34 | 8.4 | 120 | 27.1 | 0.0 | 2.4 | 0.80 | 0.34 | 0.98 |
| Mean | | | | 31 | 5.9 | 82 | 23.1 | -2.0 | 1.4 | 0.35 | 0.11 | 0.46 |
| Median | | | | 31 | 6.4 | 90 | 22.9 | -2.1 | 1.3 | 0.34 | 0.10 | 0.43 |
| Standard deviation | | | | 2 | 1.7 | 24 | 2.8 | 1.2 | 0.4 | 0.13 | 0.07 | 0.18 |
|  |  |  |  |  |  |  |  |  |  |  |  |  |
| **October-2023** | | | |  |  |  |  |  |  |  |  |  |
| 1 | 7.425 | 80.4598 | River | 27 | 8.4 | 109 | 25.2 | -4.9 | 1.4 | 0.33 | 0.139 | 0.5 |
| 2 | 7.4552 | 80.5248 | River | 27 | 8.4 | 109 | 24.9 | -5.7 | 1.6 | 0.23 | 0.124 | 0.4 |
| 3 | 7.5621 | 80.4973 | River | 26 | 7.3 | 94 | 25.0 | -6.1 | 2.1 | 0.4 | 0.104 | 0.5 |
| 4 | 7.5906 | 80.3665 | River | 29 | 8.4 | 108 | 25.1 | -6.3 | 1.6 | 0.39 | 0.110 | 0.5 |
| 5 | 7.6542 | 80.5041 | River | 29 | 7.8 | 101 | 25.0 | -6.2 | 1.3 | 0.28 | 0.150 | 0.4 |
| 6 | 7.6781 | 80.3701 | River | 29 | 6.3 | 81 | 25.4 | -5.7 | 2.1 | 0.64 | 0.093 | 0.7 |
| 7 | 7.7714 | 80.4456 | Reservoir | 31 | 7.9 | 102 | 17.6 | -1.2 | 1.0 | 0.57 | 0.131 | 0.7 |
| 8 | 7.741 | 80.3801 | River | 29 | 5.8 | 75 | 26.2 | -5.1 | 2.4 | 0.75 | 0.094 | 0.8 |
| 9 | 7.6996 | 80.3072 | Reservoir | 29 | 8.3 | 107 | 19.2 | -0.6 | 1.0 | 0.49 | 0.325 | 0.8 |
| 10 | 7.4304 | 80.333 | River | 29 | 8.3 | 95 | 25.4 | -4.6 | 1.5 | 0.62 | 0.074 | 0.7 |
| 11 | 7.4982 | 80.3842 | River | 29 | 6.7 | 86 | 25.1 | -5.0 | 2.0 | 0.78 | 0.229 | 1.0 |
| 12 | 7.547 | 80.3068 | River | 28 | 7.1 | 95 | 25.7 | -4.2 | 3.8 | 0.72 | 0.007 | 0.7 |
| 13 | 7.6266 | 80.2935 | River | 26 | 6.8 | 95 | 26.1 | -4.3 | 2.8 | 0.66 | 0.138 | 0.8 |
| 14 | 7.6705 | 80.2877 | Reservoir | 31 | 8.3 | 109 | 21.1 | -3.4 | 0.6 | 0.68 | 0.267 | 0.9 |
| 15 | 7.7179 | 80.274 | Reservoir | 29 | 9.1 | 124 | 20.1 | -1.0 | 0.7 | 0.5 | 0.000 | 0.5 |
| 16 | 7.7314 | 80.2427 | Reservoir | 28 | 9.6 | 124 | 18.3 | -1.1 | 0.9 | 0.52 | 0.183 | 0.7 |
| 17 | 7.8301 | 80.1398 | Reservoir | 28 | 5.1 | 66 | 23.0 | -1.7 | 1.3 | 0.59 | 0.045 | 0.6 |
| 18 | 7.7778 | 80.1066 | River | 28 | 6.6 | 85 | 26.4 | -2.9 | 3.5 | 1.04 | 0.323 | 1.4 |
| 19 | 7.7468 | 80.1035 | River | 29 | 4.6 | 59 | 24.0 | -4.2 | 1.6 | 0.22 | 0.233 | 0.5 |
| 20 | 7.747 | 80.1195 | Reservoir | 29 | 5.6 | 72 | 17.8 | -3.9 | 1.1 | 0.56 | 0.351 | 0.9 |
| 22 | 7.6954 | 80.1252 | River | 29 | 3.5 | 46 | 26.6 | -3.1 | 1.9 | 0.88 | 0.402 | 1.3 |
| 23 | 7.6977 | 79.9985 | River | 30 | 7.9 | 102 | 22.7 | -2.2 | 0.8 | 0.59 | 0.006 | 0.6 |
| 24 | 7.6495 | 80.1255 | River | 27 | 6.4 | 83 | 25.6 | -3.9 | 2.0 | 1.28 | 0.023 | 1.3 |
| 25 | 7.5494 | 80.2111 | River | 29 | 6.4 | 82 | 26.7 | -2.9 | 4.4 | 0.91 | 0.351 | 1.3 |
| 26 | 7.5755 | 80.2069 | River | 28 | 4.7 | 61 | 26.8 | -3.0 | 2.1 | 0.98 | 0.090 | 1.1 |
| 27 | 7.5557 | 80.192 | River | 29 | 6.6 | 85 | 26.3 | -3.0 | 3.9 | 0.96 | 0.001 | 1.0 |
| 28 | 7.615 | 80.0839 | River | 29 | 6.5 | 84 | 26.5 | -3.3 | 4.2 | 0.91 | 0.009 | 0.9 |
| 29 | 7.6378 | 80.0241 | River | 29 | 4.4 | 56 | 27.7 | -3.8 | 2.5 | 0.81 | 0.129 | 0.9 |
| 30 | 7.6192 | 79.9066 | River | 31 | 7.5 | 96 | 22.8 | -3.2 | 1.0 | 0.8 | 0.068 | 0.9 |
| 31 | 7.6041 | 79.8125 | Reservoir | 30 | 6.6 | 86 | 22.1 | -3.6 | 1.2 | 0.75 | 0.037 | 0.8 |
|  |  |  |  |  |  |  |  |  |  |  |  |  |
| Minimum | | | | 26 | 3.5 | 46 | 17.6 | -6.3 | 0.6 | 0.22 | 0.00 | 0.36 |
| Maximum | | | | 31 | 9.6 | 124 | 27.7 | -0.6 | 4.4 | 1.28 | 0.40 | 1.36 |
| Mean | | | | 29 | 6.9 | 89 | 24.0 | -3.7 | 1.9 | 0.66 | 0.14 | 0.80 |
| Median | | | | 29 | 6.8 | 90 | 25.1 | -3.7 | 1.6 | 0.65 | 0.12 | 0.79 |
| Standard deviation | | | | 1 | 1.5 | 19 | 2.9 | 1.6 | 1.1 | 0.25 | 0.12 | 0.27 |

**Figure S2.** Correlations between R/P ratios and δ^18^O_DO_ values of surface water samples in the Deduru Oya River.

**References**

92. Sander, R. Compilation of Henry’s law constants (version 4.0) for water as solvent. Atmospheric Chemistry and Physics 15, 4399–4981 (2015).

93. Sander, R. Compilation of Henry’s law constants (version 5.0.0) for water as solvent. Atmospheric Chemistry and Physics 23, 10901–12440 (2023).
